# Micromechanical Modeling of AlSi10Mg Processed by Laser-Based Additive Manufacturing: From as-Built to Heat-Treated Microstructures

**DOI:** 10.3390/ma15165562

**Published:** 2022-08-13

**Authors:** Aravindh Nammalvar Raja Rajan, Marcel Krochmal, Thomas Wegener, Abhishek Biswas, Alexander Hartmaier, Thomas Niendorf, Ghazal Moeini

**Affiliations:** 1 Institute of Mechanical Engineering, Westphalian University of Applied Sciences, Neidenburger Straße 43, 45897 Gelsenkirchen, Germany; 2 Institute of Materials Engineering—Metallic Materials, University of Kassel, Mönchebergstraße 3, 34125 Kassel, Germany; 3 VTT Technical Research Centre of Finland Ltd., Vuorimiehentie 2, FI-02150 Espoo, Finland; 4 Interdisciplinary Centre for Advanced Materials Simulation (ICAMS), Ruhr-Universität Bochum, Universitätsstr 150, 44801 Bochum, Germany

**Keywords:** laser-based powder bed fusion of metals, heat treatment, crystal plasticity, J2 plasticity, tensile behavior, nanoindentation

## Abstract

The unique microstructure of the alloy AlSi10Mg produced by the laser-based powder bed fusion of metals (PBF-LB/M) provides high-strength and high-strain-hardening capabilities of the material. The microstructure and mechanical properties of 3D-printed, i.e., additively manufactured, AlSi10Mg are significantly altered by post-building heat-treatment processes applied in order to tailor the final properties of the parts. Using an accurate computational model to predict and improve the mechanical performance of 3D-printed samples considering their microstructural features can accelerate their employment in envisaged applications. The present study aims to investigate the correlation between microstructural features and the mechanical behavior of as-built, direct-aged, and T6 heat-treated samples of PBF-LB/M AlSi10Mg under tensile loading using experiment and microstructure-sensitive modeling approaches. Nanoindentation tests are used to calibrate the parameters of the constitutive models for the Al and Si-rich phases. The experimental investigations revealed that heat treatment significantly changes the sub-grain morphology of the Si-rich phase, and this can have a considerable effect on the mechanical behavior of the components. The effect of the modeling of the Si-rich phase in the representative volume elements on the prediction of mechanical behavior is investigated using the J2 plasticity model. The combination of the crystal plasticity model for Al and the J2 plasticity model for the Si-rich phase is used to predict the tensile properties of the as-built and heat-treated states. The predicted results are in good agreement with the experimental results. This approach can be used to understand the microstructure–property relationship of PBF-LB/M AlSi10Mg and eventually tailor heat treatment for PBF-LB/M AlSi10Mg based on the requirement of the application.

## 1. Introduction

Laser-based powder bed fusion (PBF-LB/M), an additive manufacturing (AM) technique that produces a metal part by selectively melting metal powder within a powder bed using a high-power laser beam, is gaining a lot of attention from numerous industries due to its ability to produce complex, light-weight parts being characterized by unprecedented degrees of design freedom. Due to the complex thermal cycles consisting of repeated melting and rapid solidification, PBF-LB/M promotes the evolution of parts with a hierarchical microstructure. This is not possible in conventional manufacturing, thereby giving the PBF-LB/M parts better mechanical properties compared to their conventionally manufactured counterparts in case the specific boundary conditions are adequately considered.

The inherited complex thermal history during the PBF-LB/M process leads to the formation of an ultrafine supersaturated Si-rich network inside each grain of AlSi10Mg, which provides high-strength and strain-hardening capabilities [1], as well as sufficient toughness [2] to the alloy. The microstructure and the mechanical properties of the PBF-LB/M AlSi10Mg part are dependent on the process parameters, which, depending on the part’s geometry and powder properties, must be optimized to obtain defect-free microstructures. However, PBF-LB/M AlSi10Mg also presents shortcomings that simply cannot be eliminated by the optimization of the process parameters [3,4]. The defects associated with PBF-LB/M, such as porosity [5] and microstructural inhomogeneity [6,7], affect the ductility and fatigue resistance [1,8] of PBF-LB/M AlSi10Mg. Post-AM heat-treatment processes are applied to modify the microstructure of PBF-LB/M AlSi10Mg and to improve various mechanical properties. The Si, arranged in the network form around α-Al in an as-built condition [9], changes into a globular form, and the residual stresses induced during the building process are already relieved during annealing at low temperatures (300 °C) [10]. The same applies to the solution treatment at a relatively high temperature (500 °C) [10,11,12]. However, concomitantly, the ductility and fatigue resistance of PBF-LB/M AlSi10Mg are improved [4,10]. Alternatively, friction stir processing (FSP) improves the ductility [13,14,15] and fatigue life [13] of PBF-LB/M AlSi10Mg by homogenizing the microstructure and eliminating AM-induced material defects.

Understanding the microstructure–property relationships in the case of PBF-LB/M as-built (AB) and heat-treated (HT) counterparts is crucially important: the exact knowledge of the deformation behavior and damage mechanisms is paramount to be able to tailor the microstructure according to the requirements set by the applications. Eventually, this allows us to fully exploit the layer-by-layer manufacturing principle being characteristic of the PBF-LB/M process. Despite numerous experimental investigations on PBF-LB/M AB [3,16,17,18,19] and post-processed [4,10,11,12,13,14,15,20] parts, only little attention has been paid to the development of a microstructure sensitive numerical modeling approach being able to capture the effect of the microstructural features and defect distribution of the PBF-LB/M AB and HT parts on the micromechanical deformation and failure behavior.

Taheri Andani et al. [21] used a micromechanical modeling computational approach to evaluate the effect of process-induced defects, mechanical loading direction, and the role of texture on the mechanical behavior of PBF-LB/M 316L parts. Prithivirajan et al. [22] developed a crystal plasticity finite element model (CPFEM) to simulate the effect of critical pore size on the fatigue behavior of PBF-LB/M IN718, and they revealed that the approach proposed can be exploited to identify process build parameters to mitigate pores larger than the critical size. Ahmadi et al. [23] used CPFEM and a cohesive zone model (CZM) to study the effect of laser parameters, scan strategy, porosity, and grain orientations on the mechanical properties of PBF-LB/M 316L stainless steel. Some of the authors of the current research studied the effect of the microstructural features on the hardening behavior and the effect of grain statistics on the micromechanical modeling of PBF-LB/M 316L stainless steel using the CPFEM [24,25].

Kim et al. [26] used CPFEM to predict the tensile behavior of PBF-LB/M AlSi10Mg. An artificial microstructure with randomly distributed Si particles was generated. Each element was assumed to be a grain, and the orientation distribution function (ODF) of each phase extracted from the filtered EBSD data was assigned to the elements of that phase. The crystal plasticity (CP) parameters of Al and Si were obtained by fitting the macroscopic stress response and the lattice strain, experimentally determined using an in-situ neutron diffraction method. The comparison with the experimental results confirmed the accuracy of the developed computational model.

Zhang and Andrä [27] used crystal plasticity fast-Fourier transformation (CPFFT) to determine the effect of hardening parameters of Al, the aspect ratio of the Si particles, and the volume fraction of porosity to predict the tensile behavior of PBF-LB/M AlSi10Mg. The experimental results obtained from an in-situ synchrotron X-ray diffraction study [19] were used to determine the volume fraction of the Si and to calibrate the material and geometrical parameters. Using these parameters, the interphase and intergranular stress and strain distributions of PBF-LB/M AlSi10Mg under the tension were determined with the help of a 3D representative volume element (RVE) using CPFFT. The results clearly reveal that the microscopic strain and stress fields of Al and the microscopic stress distribution related to Si are inhomogeneous. Zhang et al. [28] investigated the fatigue behavior of PBF-LB/M AlSi10Mg using CPFEM, and predicted the fatigue life using Morrow’s mean stress correction and Smith–Watson–Topper models under very-high-cycle fatigue loadings. The authors concluded that the pores had a more pronounced influence on strain localization and fatigue life than inclusions.

Even though preliminary micromechanical simulations have been performed on PBF-LB/M AlSi10Mg to determine its tensile [26,27] and fatigue properties [28], and despite the fact that numerous experimental studies [4,10,11,12,13,14,15,20] reporting on HT conditions of the PBF-LB/M AlSi10Mg are available in the literature, there is still no study reporting the micromechanical deformation and damage using numerical simulations on the HT conditions of PBF-LB/M AlSi10Mg. Thus, the present work has been conducted to quantitatively model the effect of PBF-LB/M AlSi10Mg AB and HT microstructures on the tensile properties by considering all the microstructural features of the respective conditions using a micromechanical model. In the present work, the effect of modeling the Si-rich phase in the RVE of the AB state is investigated using a J2 plasticity model. Two modeling methods, one containing the J2 plasticity model for both the Al and Si-rich phases and the other a hybrid method, containing the crystal plasticity model for Al and the J2 plasticity model for the Si-rich phase are used in this study. The results from both the modeling methods are compared with the experimental results to provide an assessment on the validity of the applied models to predict the mechanical behavior of PBF-LB/M AlSi10Mg as-built and heat-treated microstructures.

## 2. Experimental Procedure

### 2.1. Material Processing

The chemical composition of the AlSi10Mg powder used for 3D printing is shown in Table 1. Specimens for microstructural characterization were manufactured by PBF-LB/M using a commercially available SLM280^HL^ machine by SLM Solutions GmbH (Lübeck, Germany). The machine was equipped with both a 400 W and a 1000 W fiber laser. For the present study, only the 400 W Yb:YAG laser was applied to manufacture AlSi10Mg specimens with a size of 200 × 50 × 6 mm^3^ under an inert argon gas atmosphere. The laser scanning vectors were set to a 45° angle (with respect to the longitudinal specimen side) in order to keep scan track lengths similar throughout the cross-section. A laser power of 400 W was used in combination with a scan speed of 1100 mm/s, hatching distance of 0.2 mm, a layer thickness of 60 μm, and substrate plate temperature of 100 °C. Scan vectors were rotated by 90° for every layer.

In order to achieve different microstructural conditions, two heat treatments were considered in the present study. First, the as-built condition was subjected to a direct aging (DA) treatment at 300 °C for two hours. Furthermore, some specimens were subjected to T6 treatment, consisting of a solutionizing step at 560 °C for 2 h, followed by water quenching and immediate artificial aging at 180 °C for 12 h. In the remainder of this paper, the DA and T6 specimens are referred to as HT1 and HT2 states, respectively.

### 2.2. Mechanical Testing

**Figure 1:**
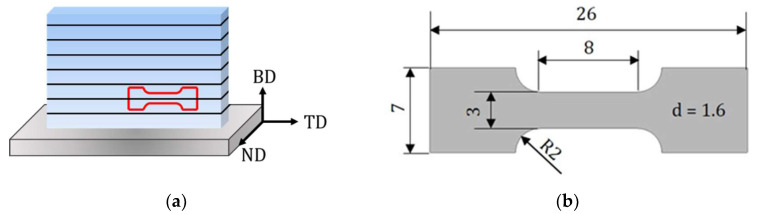
(**a**) Schematic illustration of the sample produced by PBF-LB/M. The superimposed schematic highlights the sample used for the mechanical testing. (**b**) Specimen geometry used for mechanical testing. The loading axis is oriented perpendicular with respect to building direction (BD), as seen in the schematic representation. (TD—transverse direction, ND—normal direction).

For mechanical testing, flat, dog-bone-shaped specimens with a nominal gauge section of 8 × 3 × 1.6 mm^3^ were electro-discharge machined (EDM) (cf. Figure 1) with the loading axis oriented perpendicular to the building direction (BD). The specimen geometry employed has been widely used in the literature (e.g., [29,30,31,32]). However, it has not been captured by an official standard. In order to remove the surface layer affected by EDM, specimens were subsequently ground down to a grit size of P1200 using silicon carbide grinding paper before testing. Tensile tests were conducted using an electromechanical MTS criterion testing system in displacement control with a constant crosshead speed of 2 mm/s. Strains were measured using a 5 mm gauge-length MTS extensometer directly attached to the specimen’s surface.

Microstructural analysis comprising secondary electron (SE) and electron backscatter diffraction (EBSD) maps was conducted using a Zeiss ULTRA GEMINI (Oberkochen, Germany) high-resolution scanning electron microscope (SEM) operated at an acceleration voltage of 20 kV. For EBSD measurements, a pixel size of 2 µm was used. The obtained EBSD data were analyzed using MTEX [33]. The mechanical characterization of individual phases was performed using an Anton Paar NHT^3^ Nanoindentation tester equipped with a Berkovich indenter tip. The maximum loading forces used were 0.7 mN and 0.5 mN for the as-built and HT1 conditions, respectively. Dwell times at peak load were set to 5 s. For all the aforementioned measurements, specimens were mechanically ground down to a grit size of P4000 and subsequently vibropolished for 16 h using a 0.06 μm amorphous colloidal silica suspension (Buehler MasterMet, Leinfelden-Echterdingen, Germany).

### 2.3. Microstructure Characterization

**Figure 2:**
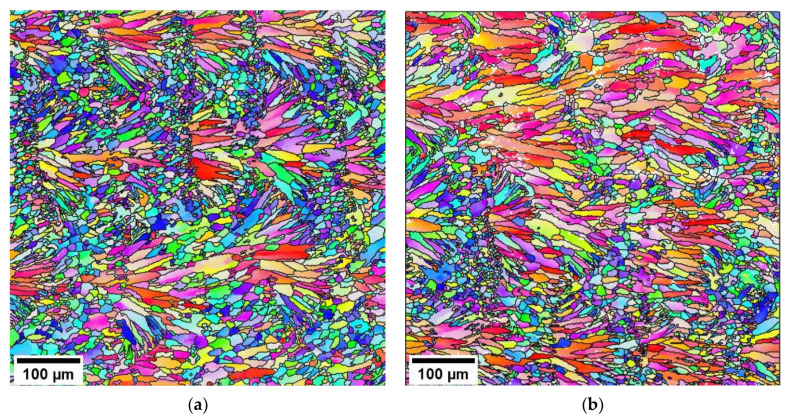

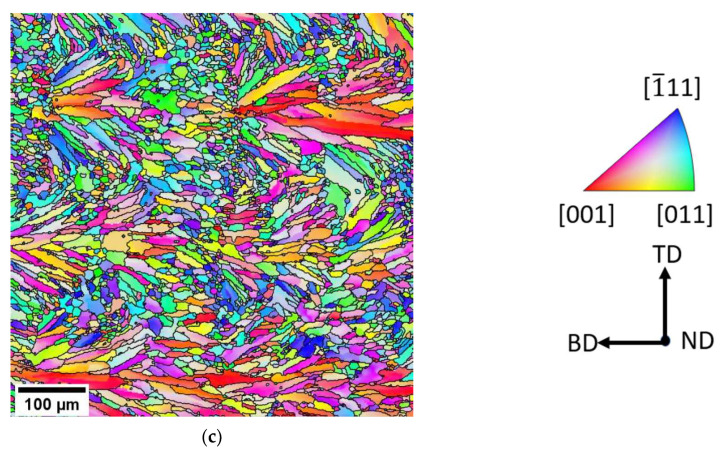
EBSD inverse pole figure (IPF) maps along x-direction (IPF-X), i.e., along BD of PBF-LB/M AlSi10Mg: (**a**) AB, (**b**) HT1, and (**c**) HT2 conditions.

**Figure 3:**
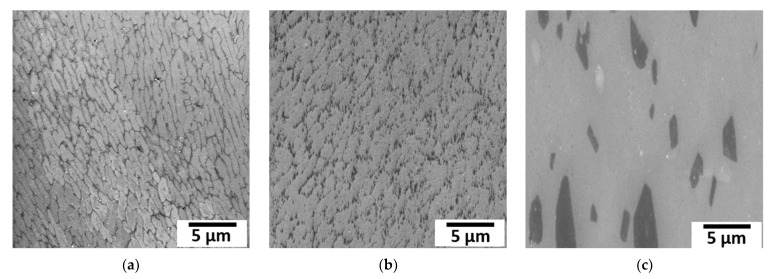
SEM images of PBF-LB/M AlSi10Mg: (**a**) AB, (**b**) HT1, and (**c**) HT2 conditions.

Figure 2 shows representative EBSD inverse pole figure (IPF) maps along the BD (x-direction) of PBF-LB/M AlSi10Mg AB and HT states. The EBSD map of the AB state, as depicted in Figure 2a, shows the presence of large columnar grains with preferred <001> texture along the BD, accompanied by some equiaxed grains with no preferred orientation. Generally, the solidification morphology is determined by the solidification parameter G/R at the solid–liquid interface, where G and R represent the thermal gradient and solidification rate, respectively. According to Liu et al. [34], planar, cellular, columnar dendritic, and equiaxed dendritic are formed in the sequence of decreasing values of G/R. Due to the high value of the solidification parameter G/R, the high quantity of grains nucleated during the melting of the powder and the base metal competitively grow to columnar grains. However, only some grains, being characterized by specific orientations (preferentially grains with 〈001〉 orientation parallel to the scanning direction), grow towards the melt pool center, whereas other grains, being characterized by random crystallographic orientation, remain as equiaxed grains at the border of the melt pool [34]. In contrast, the development of equiaxed grains can also be observed at the top of the melt pool as a result of the so-called columnar to equiaxed transition (CET). During PBF-LB/M processing, CET eventually occurs when the nucleation of sufficiently numerous equiaxed grains occurs in the undercooled liquid adjacent to the columnar dendritic front. As a result of the overlap between neighboring tracks, the partial re-melting of previous layers can be observed, and therefore only a small part of the equiaxed grains formed by CET remains. For a detailed explanation of the CET and solidification, the reader is referred to the literature [34,35,36,37]. The sub-grain microstructure being characteristic of the PBF-LB/M AB condition known from the literature [4,9,38] with a thin, continuous Si-rich network structure around the α-Al cells is clearly visible in Figure 3a, depicting a micrograph obtained by SEM for the AB state. During the PBF-LB/M process, α-Al solidifies with a columnar morphology with continuous segregations of inter-dendritic Si at the boundaries along BD. When viewed from the plane parallel to the transverse direction (TD) and normal direction (ND), the columnar or elongated Al cells are observed to be equiaxed [9,39]. Wu et al. [39] concluded that these equiaxed Al cells are the cross-section of the columnar Al cells observed alongside BD. Only a small fraction of Si and Mg (on the nanometer scale) are dispersed within the Al cells [40]. Based on the analysis of results obtained by high-resolution transmission electron microscopy and energy dispersive spectrometer (EDS), Chen et al. [1] determined that the Si-rich network was made of alternate eutectic Al and Si phases. The fraction of Si in the Al cells and Al in the Si-rich network depends on the building parameters applied in the PBF-LB/M process. The Si-rich networks surrounding Al in AB state act as an obstacle to dislocation movement. Thus, these structures are responsible for the higher strength of PBF-LB/M AlSi10Mg AB components.

The EBSD maps of the PBF-LB/M AlSi10Mg HT states, as illustrated in Figure 2b,c, also show the presence of large columnar grains and some equiaxed grains with no specific preferred orientation. The heat treatment of the PBF-LB/M AB state only causes a minor coarsening of the microstructure. Average grain diameters of 17.7 µm and 16.4 µm for the HT1 and HT2 conditions, respectively, are hardly affected when directly compared to the average grain diameter of 15.8 µm for the AB state. The heat treatment has a much more pronounced effect on the sub-grain microstructure. As already reported in the previous studies [4,10,12], during the heat treatment processes, the Si-rich network breaks up and changes into a globular form. As can be deduced from Figure 3b, after HT1, the Si-rich structure is broken up into small individual Si-rich particles. These particles are arranged in a pattern resembling a discontinuously structured network around the Al-cells. A change in the heat treatment to the T6 treatment used for HT2 eventually coarsens these Si particles significantly, as can be deduced from Figure 3c. The particles tend to form angular shapes with diameters of up to several micrometers. These are distributed more or less evenly across the Al matrix (cf. Figure 3c). Comparable results were also reported by Alghamdi et al. [41], employing a T6 treatment, including solutionizing at 520 °C for 1 h, water quenching, and artificial aging at 170 °C for 4 h. The authors showed irregular-shaped Si particles with sizes ranging from approximately 200 nm to 4 μm. According to the observations of Van Cauwenbergh et al. [40], there was a change in the amount of Si in the Al cells and Al in the Si-rich particles during the heat treatment. These changes in chemical composition were found to be dependent on the process parameters of the heat-treatment and PBF-LB/M processes, respectively. Therefore, only minor local changes in particle properties across the different specimen conditions were to be expected in the present study.

## 3. Generation of Synthetic Microstructure

A synthetic microstructure used for the micromechanical modeling approach must contain all the essential microstructural features and must be statistically equivalent to the experimentally determined microstructure [24] to maintain the representativeness of the material considered. Therefore, the RVE must consider the morphology and the volume fraction of the Si-rich phase and consider the grain morphology and the crystallographic texture specific to the AB and HT conditions. To study the effect of the modeling approach considered for the Si-rich phase in the AB state, two sub-grain RVEs were created from the SEM micrographs obtained in the experiments (cf. Figure 3). Furthermore, to investigate the effect of heat treatment on PBF-LB/M AlSi10Mg, an RVE with multiple grains was created for each state from the corresponding EBSD maps (namely, mesoscale RVEs), considering the proper statistics of grain geometries and crystallographic orientations. The following subsections describe in detail the methods and parameters used for generating the different RVEs.

### 3.1. RVE from SEM

**Figure 4:**
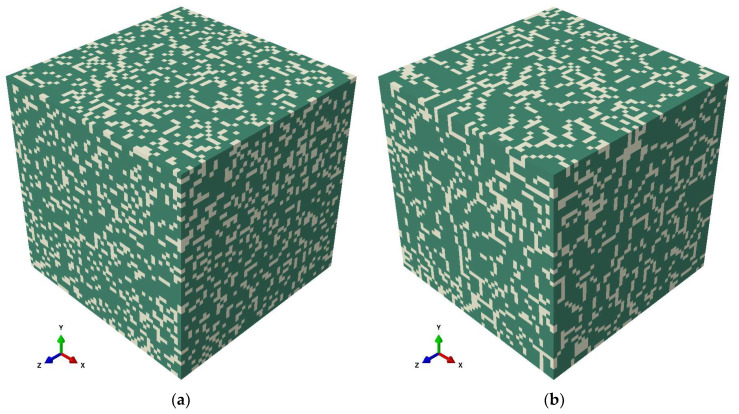
Three-dimensional synthetic RVEs generated from the experimentally determined SEM image, where the green region represents the Al matrix, and the yellow regions indicate regions of the Si-rich phase. (**a**) AB RVE with random Si-rich phase (random RVE), and (**b**) AB RVE with network-patterned Si-rich phase (network RVE).

The major and minor axes of the Al cells, the thickness of the Si-rich network of the AB state, and the volume fraction of the Si-rich phase were deduced from the SEM image using the software package ImageJ 1.53e (National Institutes of Health, Bethesda, MD, USA) [42]. Two sub-grain scale RVEs with the size of 5 µm × 5 µm × 5 µm with random and network-patterned structures were created for the AB condition. The first RVE of the AB state (referred to as random RVE) was constructed using ABAQUS [43] and meshed using 3D solid elements, C3D8, with the element size equal to the thickness of the Si-rich network in the SEM image. The Si-rich particles were then randomly distributed considering the volume fraction using a python script (see Figure 4a).

In the AB state, the Si-rich phase forms a network around elongated Al cells observed along BD, which are approximately equiaxed along ND and TD [39]. For the second RVE of the AB state, the Al cells were considered as spheroidal cells surrounded by the Si-rich network (see Figure 4b). From the major and minor axes measurements, the logarithmic normal values of the equivalent diameter and the aspect ratios were calculated using MATLAB [44]. A 3D synthetic microstructure was created in the software package DREAM.3D [45] using the logarithmic normal values. The boundary elements between two cells were assigned to the Si-rich phase based on the volume fraction using a python program. This allowed for the creation of the RVE (named network RVE) with the required volume fraction and network structure as depicted in Figure 4b.

### 3.2. RVE from EBSD

The thermal history during the PBF-LB/M process influences the grain geometry as well as the grain orientations. To capture the anisotropic behavior of PBF-LB/M materials, the RVE must mimic the grain geometry and the crystallographic texture experimentally determined by EBSD. For generating a statistically equivalent RVE, the EBSD data used must contain a minimum of 10,000 grains [25]. Alternatively, smaller EBSD maps can be captured at various areas and stitched together [25]. The following subsections explain the procedure for generating the mesoscale RVEs in detail.

#### 3.2.1. Microstructure Geometry

**Figure 5:**
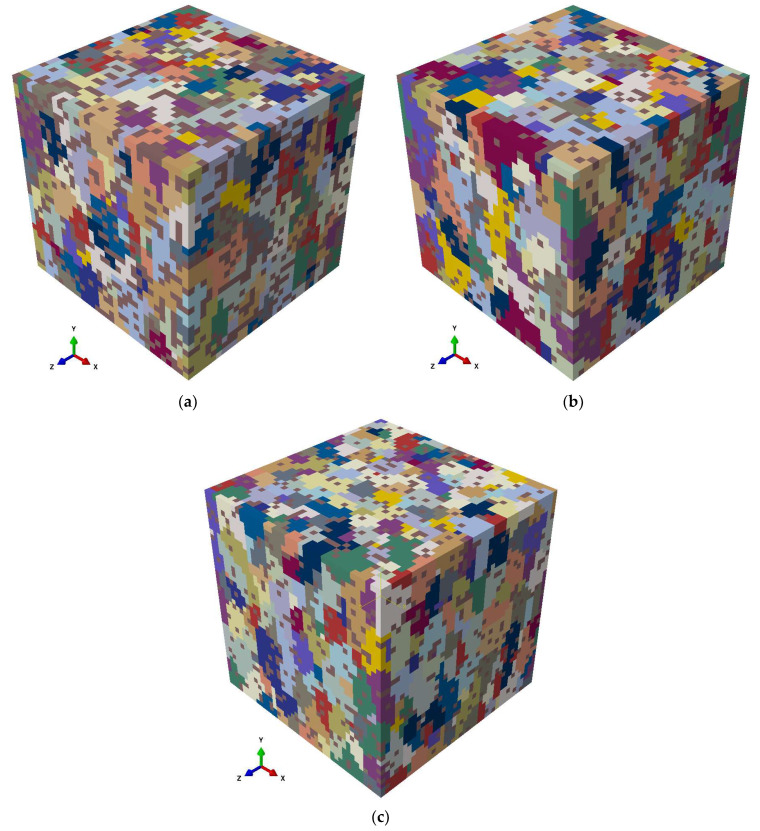
Three-dimensional synthetic mesoscale RVEs of each state generated from the EBSD data of each state using Kanapy with randomly distributed Si-rich phase (shown in brown), (**a**) AB state RVE with 503 grains and 25% volume fraction of Si-rich phase, (**b**) HT1 state RVE with 506 grains and 16.4% volume fraction of Si-rich phase, and (**c**) HT2 state with 502 grains and 11.5% volume fraction of Si-rich phase.

The grains established by the PBF-LB/M process can be approximated as ellipsoids that are elongated along BD and equiaxed along ND and TD due to the scan rotation in the subsequent layers [24]. To reduce the computational time associated with the simulation model considering the network structure, the Si-rich phase was assumed to be randomly distributed for both AB and HT states. The open-source software Kanapy [46] was used to create the RVE with ellipsoidal grains using the equivalent diameter of the sphere and the aspect ratio measured from experimentally determined EBSD with the help of MTEX [33] as inputs. Kanapy populates the RVE using voxels and assigns these voxels to the ellipsoidal particles to generate a space filling structure that resembles the experimental one in terms of grain size distribution, aspect ratio distribution of grains, and distribution of orientation angles of the major axis of the grains [25]. The generated hexahedral mesh (C3D8 elements) is compatible with ABAQUS. RVEs with a minimum of 500 grains were generated for each condition based on the statistically confirmed input data obtained from EBSD. The Si-rich secondary phase was randomly distributed inside the grains according to the volume fraction of each state, as detailed in Figure 5.

#### 3.2.2. Representative Orientations

**Figure 6:**
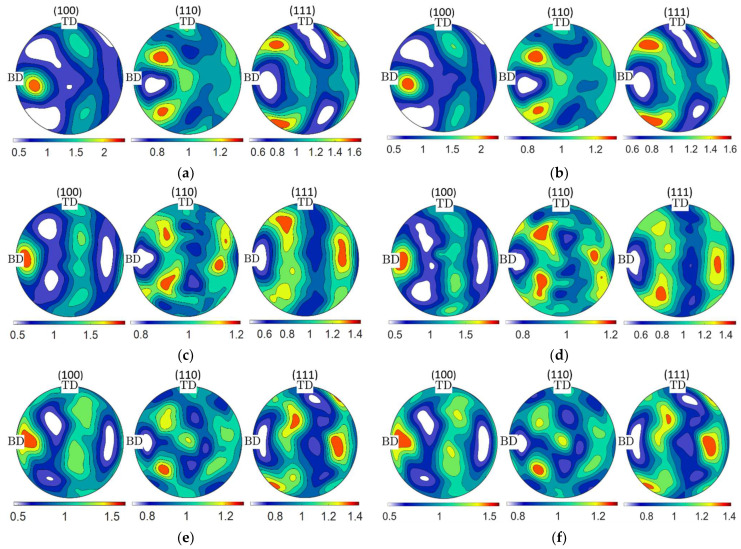
Pole figures for the ODFs estimated from the EBSD data, (**a**) AB state, (**c**) HT1 state, (**e**) HT2 state, and (**b**,**d**,**f**) reconstructed ODFs from the orientations of the AB and HT states, respectively.

Crystallographic texture plays a significant role in the mechanical behavior of the polycrystalline material, as it determines the grain orientation and grain boundary misorientation angle. Therefore, to determine the tensile properties of the PBF-LB/M AlSi10Mg AB and HT conditions, the RVE texture must replicate the crystallographic texture observed in the EBSD. The orientation distribution function (ODF) can be used to represent the crystallographic texture [47]. For this purpose, the ODFs calculated from EBSD data must be reduced to a smaller number of representative orientations. Using the filtered EBSD data and the grain morphology data from MTEX as inputs, Kanapy calculates the ODF based on the de la Valeé kernel function [48]. Since the RVE is constructed with smaller variations in grain size, Kanapy extracts equally weighted orientations from the EBSD dataset using the L1 minimization scheme [49]. It can be deduced from Figure 6 that the difference between the ODFs calculated from the experimental EBSD data and the ODFs calculated from the representative orientations of the RVE is small.

**Figure 7:**
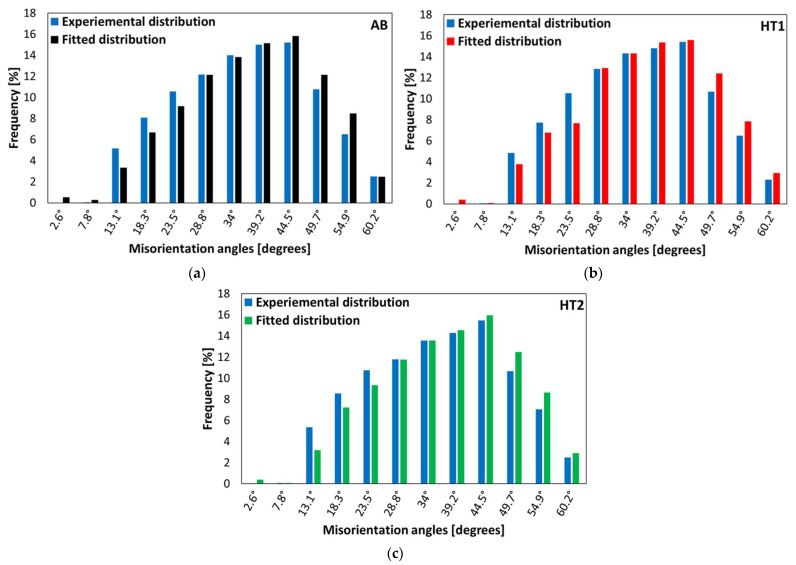
Grain boundary misorientation angle distribution obtained from the experiment and from the Kanapy fitted results, (**a**) AB, (**b**) HT1, and (**c**) HT2 states.

In addition to the crystallographic texture, polycrystalline materials also are characterized by a specific grain boundary texture, which can be described by the grain boundary misorientation angle distribution. To replicate the distribution experimentally determined by EBSD, the reduced orientations are assigned to the grains of the RVE based on the algorithm suggested by Biswas et al. [24] using Kanapy. Figure 7 clearly proves that the fitted grain boundary misorientation angle distribution is in good agreement with the experimentally determined distribution.

## 4. Micromechanical Modeling

The plastic deformation in metals is governed by the movement of dislocations in specific crystallographic slip systems [50,51]. In this regard, the orientation of the grain determines the alignment of its slip systems with respect to the loading direction resulting in anisotropic mechanical behavior [24,52,53]. The grain boundaries act as an obstacle to the dislocation movement on the active slip system [54], resulting in the pile up of dislocations. As has been generally revealed in the literature, the crystal plasticity (CP) model is an appropriate choice for modeling the constitutive behavior of metals at the scale of individual grains [55].

In the present work, the isotropic J2 plasticity model was initially used to study the influence of the Si-rich phase in the mechanical response of the AB state using sub-grain RVEs. Then, a hybrid RVE with a CP model for the Al grains in combination with a J2 model for the Si-rich phase was used to predict the tensile behavior of PBF-LB/M AlSi10Mg for AB and HT conditions. The latter approach is called micromechanical modeling because the microstructure of the material is represented in a discrete way. In the following subsections, the J2 plasticity and the crystal plasticity models are explained in detail.

### 4.1. J2 Plasticity

According to the von Mises yield criterion, plastic yielding starts when the second invariant of the deviatoric stress tensor J_2_ reaches the critical value associated with the yield strength of the material [56]. The general form of the von Mises yield function ϕ is defined as [57]:

(1) $$\phi =\sqrt{\frac{3}{2}\;\sigma^{D}:\sigma^{D}}-Y\left(\bar{\varepsilon_{p}}\right)$$

where ***σ****^D^* represents the deviatoric part of the stress tensor ***σ***, and $Y\left(\bar{\epsilon_{p}}\right)$ presents the yield strength as a function of the total plastic strain $\bar{\varepsilon_{p}}$. Accordingly, plastic yielding starts when the yield function ϕ=0. The associated flow rule is given by [57]:

(2) $$d\varepsilon_{p}=d\bar{\varepsilon_{p}}\frac{\partial \phi }{\partial \sigma }$$

where $d\bar{\epsilon_{p}}$ is derived from the fact that the yield criterion must always be satisfied during plastic straining [57]. Here, the isotropic hardening according to the Voce law was applied to depict the yield behavior and flow stress of the materials. The Voce law is given as:

(3) $$Y\left(\bar{\varepsilon_{p}}\;\right)=\sigma_{y}+K\left(1-e^{-n\bar{\varepsilon_{p}}}\;\right)$$

where $\sigma_{y}$ represents the initial yield stress, K the saturation stress, and n the Voce law exponent.

### 4.2. Crystal Plasticity

This section provides a brief overview of the CP model applied for predicting the mechanical response of the Al grains in the present work. For a detailed description of the CP method, the reader is referred to studies by Ma and Hartmaier [58] and Roters et al. [59]. We followed the standard formulations, in which the total deformation gradient is multiplicatively decomposed into elastic ($F^{e}$) and plastic ($F^{p}$) components, which denotes the reversible and irreversible lattice deformations, respectively:

(4) $$F=F^{e}F^{p}$$

The plastic velocity gradient, which is a function of the plastic deformation gradient and its rate, can be used to characterize the evolution of the plastic deformation:

(5) $$L^{p}=\dot{F^{p}}F^{p-1}$$

Since the plastic deformation occurs due to the movement of dislocations along the active slip systems, the plastic velocity gradient can also be expressed as the sum of the shear rates of all contributing slip systems:

(6) $$L^{p}=\overset{N_{s}}{\underset{\alpha =1}{\sum }}{\dot{\gamma }}^{\alpha }d^{\alpha }\;⊗\;n^{\alpha }=\;\overset{N_{s}}{\underset{\alpha =1}{\sum }}{\dot{\gamma }}^{\alpha }M^{\alpha }$$

where ${\dot{\gamma }}^{\alpha }$ is the plastic shear rate for the slip system *α*; ***d**^α^* and ***n****^α^* are the slip direction and the slip plane normal of the slip system α, respectively; N_s_ denotes the number of active slip systems; and ***M****^α^* is the Schmid tensor of the slip system α. The Schmid tensor ***M****^α^* denotes the projection to the slip plane and into the slip direction, and is defined by the dyadic product of the vectors describing the slip direction (***d****^α^*) and normal to the slip plane (***n****^α^*).

Furthermore, the phenomenological constitutive model, where the shear rate is formulated as the function of the resolved shear stress $\tau^{\alpha }$ and the slip resistance ${\hat{\tau }}^{\alpha }$, was used in this work.

(7) $${\dot{\gamma }}^{\alpha }={\dot{\gamma }}_{0}{\left|\frac{\tau^{\alpha }}{{\hat{\tau }}^{\alpha }}\right|}^{p_{1}}sgn\left(\tau^{\alpha }\right)$$

${\dot{\gamma }}_{0}$ represents the reference shear rate, and the stress exponent $p_{1}$ is related to the inverse of the strain rate sensitivity. The resolved shear stress $\tau^{\alpha }$, which is the mapped stress due to the elastic strain on the slip systems, is given as:

(8) $$\tau^{\alpha }=\frac{\mathbb{C}}{2}\left(F^{eT}F^{e}-I\right)\cdot M^{\alpha }$$

Here, ℂ represents the stiffness matrix and ***I*** is the identity tensor. The hardening behavior of the material is described by the evolution of the slip resistance ${\hat{\tau }}^{\alpha }$ as:

(9) $$\dot{{\hat{\tau }}^{\alpha }}=\overset{N_{s}}{\underset{\beta =1}{\sum }}h_{0}\chi_{\alpha \beta }{\left(1-\frac{{\hat{\tau }}^{\alpha }}{{\hat{\tau }}^{f}}\right)}^{p_{2}}\left|{\dot{\gamma }}^{\beta }\right|$$

where $h_{0}$ denotes the hardening rate; $\chi_{\alpha \beta }$ is the cross-hardening matrix, in which the coplanar slip systems are represented in the diagonal elements with the values set to 1.0; and the noncoplanar slip systems are represented in the off-diagonal elements with the values set to 1.4. In addition, ${\hat{\tau }}^{f}$ is the saturation slip resistance related to the accumulation of the dislocation, and $p_{2}$ is a fitting parameter.

### 4.3. Identification of Material Parameters

For multiphase materials, such as AlSi10Mg, the behavior of each constituent in the microstructure must be characterized separately to extract its material parameters for the numerical simulations. Nanoindentation is a widely used method for extracting the material parameters of each phase of the multiphase materials. The general feasibility of obtaining true material parameters from the inverse analysis of indentation results has been demonstrated by Schmaling and Hartmaier [60] for conventional hardness tests. In more recent work, the reliability of this inverse method has been shown for nanoindentation [61] and also for cyclic indentations [62]. Thus, nanoindentation tests were performed on the Al and Si-rich phases of the AB and HT states using a Berkovich indenter. Multiple indentations were performed to account for the scatter being induced by the influence of one phase on the indentation result of the other phase, this being a direct consequence of the locally differing size and morphology of the prevailing phases. For Al, the force-displacement curve closest to the average of all the Al curves was chosen, together with the crystallographic orientation of the corresponding grain for parameter optimization for both J2 and CP models. For the Si-rich phase, the average curve was used for parameter extraction for the J2 plasticity model. Since the influence of the relatively large Si-rich phase located directly underneath the surface on the Al nanoindentation curves could not be neglected for the HT2 state, the material parameters from the HT1 state were used for the tensile simulation of the HT2 state.

The finite element simulations of the nanoindentation test were performed in ABAQUS using the built-in J2 plasticity model and using the user-defined subroutine (UMAT) for the CP model. For the application of the J2 model, Young’s modulus E and three Voce law parameters ($\sigma_{y},\;$ K and n) have to be determined. The Poisson’s ratio of Al and Si-rich phases were considered to be 0.31 and 0.33 [63], respectively. For the application of the CPFEM, four plasticity parameters ($p_{1},\;{\hat{\tau }}_{0}^{\alpha }$, $h_{0}$, ${\hat{\tau }}^{f}$) must be calibrated. The three elastic constants of the CP model of Al are C_11_ = 107.3 GPa, C_12_ = 60.9 GPa, and C_44_ = 23.8 GPa [26].

**Figure 8:**
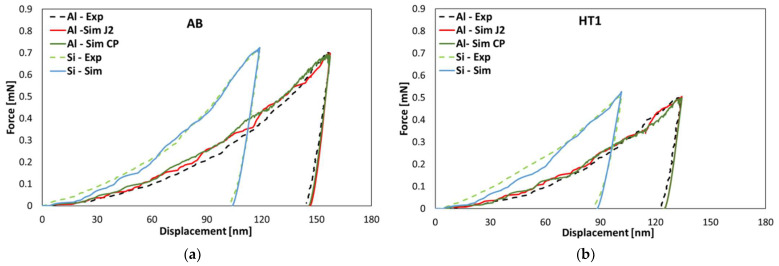
Comparison of the force-displacement curves obtained from the nanoindentation experiment as well as the J2 and CP models for Al and Si-rich phases: (**a**) AB and (**b**) HT1 states.

The material parameters to be used in the J2 and CP models were obtained by inverse fitting of the force-displacement curve obtained from the simulation with the experimental curves, as shown in Figure 8. An objective function, a function of material parameters, is required to solve the inverse analysis problem. In the present study, the mean squared error (MSE) was used as an objective function.

(10) $$MSE=\frac{1}{n}\overset{p_{e}}{\underset{p=p_{0}}{\sum }}{\left(f_{p}^{exp}-f_{p}^{sim}\right)}^{2}$$

Here, *n* is the number of data points; $p_{0}$ and $p_{e}$ are the initial and final depths, respectively; and $f_{p}^{exp}$ and $f_{p}^{sim}$ are the forces obtained from the experiment and simulation at depth *p*.

The Nelder–Mead algorithm [64,65], available in the open source python library SciPy [66], was employed for inverse analysis. The optimum material parameters were determined by the minimization of the objective function. The optimized parameters obtained for the J2 and CP models are listed in Table 2 and Table 3, respectively.

Since it is known that parameter optimization by inverse methods from indentation results may not always lead to unique results, the optimization procedure was started from different start values to make sure it always converged to the same material parameters in this case.

### 4.4. Finite Element Model

To calculate effective stress–strain curves for the different microstructures, finite element simulations were performed for boundary conditions that resulted in a uniaxial stress state. Periodic boundary conditions (PBCs) were applied because they provide a more realistic deformation of the RVE than the von Neumann and Dirichlet boundary conditions [67]. To implement the PBC, the equations based on Boeff [67] were used to constrain the respective pair of boundary nodes lying on opposite faces. Therefore, the periodicity is maintained even in the deformed state. The displacement for each condition was calculated from the experimentally observed maximum strain and the RVE size. These displacements were applied alongside TD of the RVE in ABAQUS as displacement-controlled load.

The first-order computational homogenization by volume averaging was used to establish the nonlinear meso–macro structure–property relation. The macroscopic stress and strain components were calculated from the microscopic values using the following equations [68]:

(11) $$\bar{\sigma }=\frac{1}{V_{RVE}}\int_{V_{RVE}}\underline{\sigma }\;dv$$

(12) $$\bar{\varepsilon }=\frac{1}{V_{RVE}}\int_{V_{RVE}}\underline{\varepsilon }\;dv$$

where $\bar{\sigma }$ and $\underline{\sigma }$ denote macroscopic and microscopic stresses, respectively; $\bar{\varepsilon }$ and $\underline{\varepsilon }$ denote macroscopic and microscopic strains, respectively; and V_RVE_ is the volume of the RVE.

## 5. Results and Discussion

### 5.1. Effect of Heat Treatment

**Figure 9:**
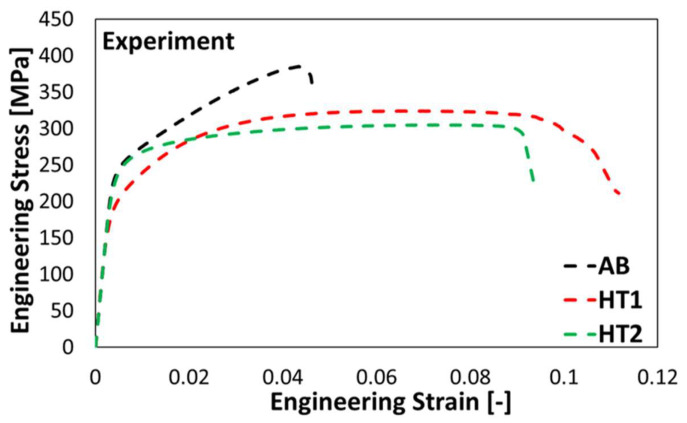
Experimental stress–strain curves of different states for PBF-LB/M AlSi10Mg obtained from uniaxial tensile tests.

The experimental investigations of PBF-LB/M in AB and HT states showed that the heat treatments had a significant effect on the strength and ductility of the material. Heat treatment of the PBF-LB/M AlSi10Mg reduces the strength of the material, whereas the ductility of the material is improved, as revealed by the results of tensile testing depicted in Figure 9. This is in line with the conclusion of previous investigations on the heat-treated conditions of PBF-LB/M AlSi10Mg [4,10].

The ultimate tensile strengths were 385 MPa, 316 MPa, and 305 MPa for AB, HT1, and HT2 states, respectively. The values were the average of five samples for the AB condition and three samples each for HT1 and HT2 conditions. The fracture occurred at absolute strains of 4.6%, 10.6%, and 9.4% for AB, HT1, and HT2 states, respectively. In the AB condition, the Si-rich network increased the strength of the material by effectively impeding the dislocation motion [1]. Dissolving the network structure then allowed for relative ease of plastic flow, due to which relatively large elongations to failure were observed in the heat-treated states. According to Van Cauwenbergh et al. [40], a conventional T6 heat treatment eliminates the positive effect of grain refinement and Si supersaturation, which are the primary strengthening mechanisms of as-built and direct aged PBF-LB/M AlSi10Mg. The experimental result obtained for the HT2 condition is in line with this conclusion. As can be deduced from Figure 9, the HT2 state has lower strength and ductility in comparison to the HT1 condition, where the latter retained some characteristics from the as-built Si-network structure (cf. Figure 3). It was concluded at this point that local instability in deformation was effectively hindered in HT1 condition.

### 5.2. Effect of Silicon-Rich-Phase Modeling

**Figure 10:**
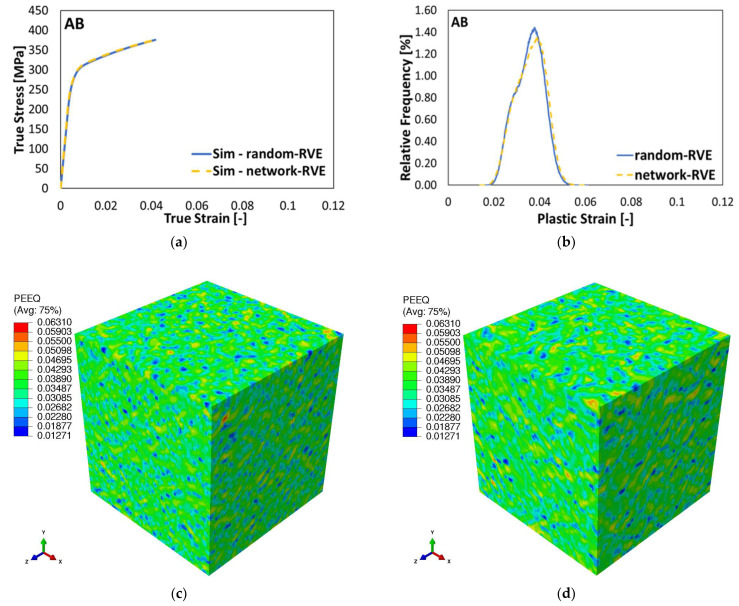
Effect of the approach used for modeling of the Si-rich phase, (**a**) macroscopic stress–strain curves, (**b**) relative frequency of the prevailing microscopic plastic strain, and (**c**,**d**) equivalent plastic strain distribution of the random RVE and network RVE, respectively.

To study the effect of the modeling of the Si-rich phase in the RVE on the robustness of prediction of the tensile properties, uniaxial tensile simulations were performed on the random RVE and network RVE using the J2 plasticity model in ABAQUS. Figure 10a compares the macroscopic stress–strain values obtained from the random RVE and network RVE. It can be clearly seen that the difference in the macroscopic stress value between the network RVE and random RVE is very negligible. A detailed study on the local microscopic plastic strain extracted at each element integration point in the form of the relative frequency is depicted in Figure 10b.

A histogram with a bin width of 0.00025 was created for the local microscopic plastic strain values, and the relative frequency was calculated using it. The relative frequency of the microscopic plastic strains in the *i*th bin ranging from $\varepsilon_{p}^{i}$ and $\varepsilon_{p}^{i}+d\varepsilon \;\left(d\varepsilon =0.00025\right)$ is

(13) $$rf^{i}=\frac{\omega^{i}}{\Omega }$$

where $\omega^{i}$ is the number of data points in the *i*th bin and Ω is the total number of data points. Figure 10b shows that there is a small difference between the random RVE and network RVE in terms of the maximum relative frequency and the plastic strain at the maximum relative frequency. Figure 10c,d show the distribution of the equivalent plastic strain in the random RVE and network RVE. A minor variation in the distribution and values of the equivalent plastic strain with respect to the morphology and the distance between the Si-rich particles can be observed in Figure 10c,d. However, these differences are very small, which contradicts the experimental observations. Various experimental studies concluded that the morphology of the Si-rich phase has a pronounced effect on the mechanical properties of Al-Si alloys [4,69]. This contradiction between the experimental observation and simulation result can be due to the limitation of the local J2 plasticity model used for the simulation. Nevertheless, it shows that the assumption of the randomly distributed Si-rich particles in the RVE presents a similar estimate of the properties of the AB condition as that of the RVE with the Si-rich network structure.

The microscopic stress and strain of the individual phases in the RVE were extracted and homogenized to obtain the macroscopic stress–strain curve of the Al and Si-rich phases. It has been determined that the homogenized stress–strain curves of the Al and Si-rich phases are located within the upper and lower limits of the results obtained by Kim et al. [63] from the lattice strain values of Al and Si obtained from the neutron diffraction measurements.

### 5.3. Validation of the Micromechanical Model

**Figure 11:**
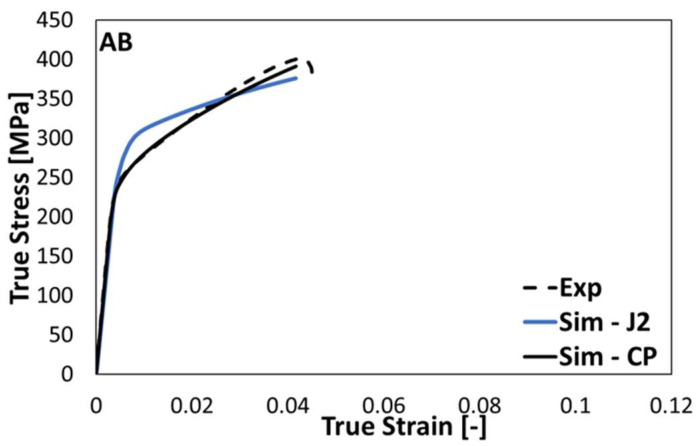
Homogenized stress–strain curves of the PBF-LB/M AlSi10Mg AB state obtained from the J2 and CP models.

Finite element simulations of uniaxial tensile tests were performed using J2 plasticity and CP models, together with the mesoscale RVEs with the help of optimized material parameters of the AB and HT1 states. The inverse procedure described above was used to determine the material parameters of the constitutive models (cf. Table 2 and Table 3). From Figure 11, it can be deduced that the stress–strain curve obtained from the CP model is in much better agreement with the experiment than that of the J2 model.

It can be observed that the CP model with parameters identified from indentation tests was able to reproduce the tensile test results without further parameter fitting, whereas the J2 plasticity model seemed to be too simple to achieve such predictive capabilities. Therefore, the crystal plasticity model was the most suitable micromechanical model to predict the mechanical properties of PBF-LB/M AlSi10Mg.

The effect of RVE size on the tensile properties of the AB state was studied using the CP model. Three RVEs of sizes 47 µm × 47 µm × 47 µm with 503 grains, 55 µm × 55 µm × 55 µm with 809 grains, and 59 µm × 59 µm × 59 µm with 1000 grains were created with the randomly distributed Si-rich phase as described earlier. The tensile simulations were performed with a combination of the J2 and CP models. It has been found that, within the range of sizes investigated, the RVE size has a negligible effect on the mechanical behavior of PBF-LB/M AlSi10Mg (result not shown), which is in agreement with the findings of Zhang et al. [27].

**Figure A1:**
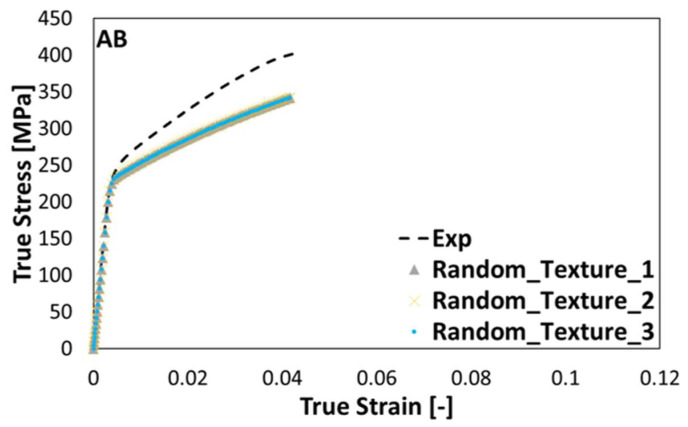
Comparison of the experimental and the predicted tensile behavior of the PBF-LB/M AlSi10Mg AB state using the RVEs with randomly generated orientations.

The effect of the texture in the RVE on the determination of the tensile properties was studied. The same RVE of the AB state was assigned with three different random orientations, and the tensile simulations were performed. A significant difference between the simulation and the experimental results is observed when random textures are used (cf. Figure A1). Therefore, the texture in RVE must mimic the experimentally observed texture to accurately predict the mechanical behavior of the PBF-LB materials. A detailed description of the procedure and result is presented in Appendix A.

**Figure 12:**
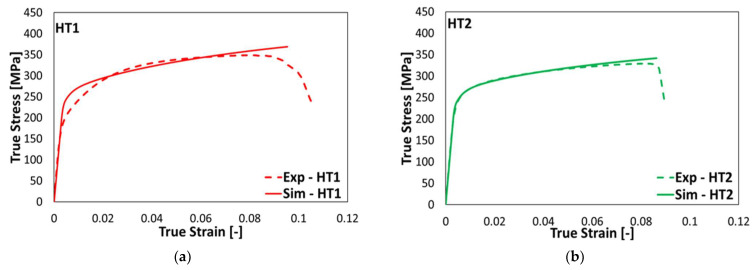
Comparison of the experimental and predicted tensile behavior using the combined crystal plasticity and J2 plasticity models of the PBF-LB/M AlSi10Mg HT states. (**a**) HT1 and (**b**) HT2 states.

Figure 12a,b present a comparison between the experimental and predicted tensile behavior of HT conditions. The CP model presents a reasonably good estimate of the tensile properties of the PBF-LB/M AlSi10Mg HT states. The minor difference in the yield region and the ultimate tensile strength may be due to the influence of one phase on the other during the nanoindentation. Since the distance between the Al and Si-rich phases is very small in AB and HT1 states, the property of one phase could be inadvertently captured during the nanoindentation test in the force-displacement curve of the other phase. It would then be reflected in the optimum material parameters obtained from the curve. However, since the difference between the experimental and the predicted results are fairly minor, the model parameters calibrated using the nanoindentation results are observed as acceptable and can therefore be used to predict the mechanical behavior of PBF-LB/M AlSi10Mg in AB and HT conditions. Similar to the AB state, the CP model presents a better estimate of the tensile behavior of the HT states compared to the J2 model.

**Figure 13:**
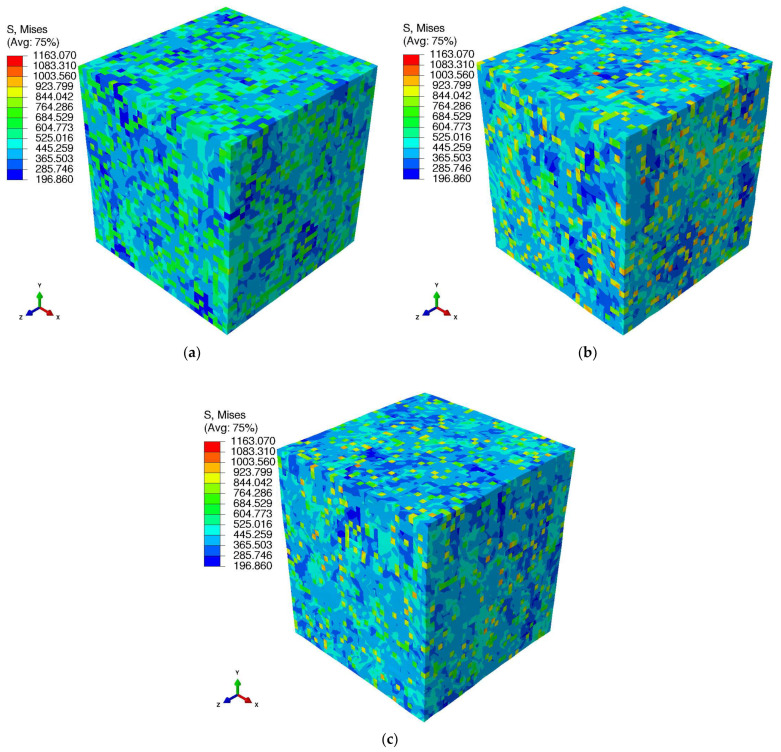
Microscopic stress field obtained using the CP model for the PBF-LB/M AlSi10Mg AB and HT states. (**a**) Stress distribution in the AB state at the applied strain of 4.25%, (**b**) stresses for the HT1 state at the applied strain of 10%, and (**c**) stresses for the HT2 state at the applied strain of 8%.

Figure 13 presents the microscopic stress fields of the AB and HT states at their respective maximum applied strain. It can be observed that the stress distributions in the Al grains and Si-rich particles are inhomogeneous for PBF-LB/M AlSi10Mg AB and HT conditions. This is due to the anisotropic behavior of the material. These inhomogeneous stress distributions of the AB state are in line with the conclusion of Zhang et al. [27].

## 6. Conclusions

In the present work, the effect of direct aging and T6 heat treatments on the microstructure of the alloy AlSi10Mg produced by laser-based powder bed fusion of metals (PBF-LB/M) was studied using experimental techniques. The influence of microstructural features of the as-built and heat-treated conditions of PBF-LB/M AlSi10Mg on tensile behavior was investigated by the combined approach of experiment and micromechanical modeling. The microstructure of the material consisted of an Si-rich phase in a polycrystalline Al matrix. The main conclusions are as follows:

1. The heat treatment of PBF-LB/M AlSi10Mg changes the morphology of the Si-rich phase from a network pattern to a globular form. This change in the morphology of the Si-rich phase renders the dislocation motion easier, thereby improving the ductility of the material at the expense of tensile strength. The ductility and tensile strength of the T6 heat-treated samples are lower than that of the direct-aged samples since T6 heat treatment completely changes the Si-rich particles to a globular form.
2. When the Si-rich phase is modeled as randomly distributed particles or as a network structure in the representative volume element (RVE), the resulting macroscopic tensile behaviors remain almost unaffected. There is only a small quantitative difference in terms of the distribution and average value of the microscopic plastic strains for the PBF-LB/M AlSi10Mg in the as-built condition.
3. Application of a micromechanical model consisting of crystal plasticity (CP) for Al grains and J2 plasticity for the Si-rich phase predicts the tensile behavior of both, as-built and heat-treated conditions, better than using merely J2 plasticity for both phases, and the results are consistent with the experimental data of tensile testing. This indicates that the CP model captures the mechanical behavior of the Al phase better than the J2 plasticity model.
4. The RVE size only has a negligible effect on the predicted results. Due to the prevailing anisotropic material behavior, an inhomogeneous microscopic stress distribution is observed for the as-built condition and all the heat-treated conditions.

## Figures and Tables

**Table 1:** Chemical composition of the AlSi10Mg alloy powder used in this study.

Element	Al	Si	Mg	Fe	Cu	Zn	Ti	Mn	Ni
Content (wt%)	Balance	9.86	0.37	0.16	0.01	0.01	0.01	0.008	<0.03

**Table 2:** Optimized J2 parameters for the Al and Si-rich phases derived by inverse analysis. See text for details.

States	Phase	Elastic Constant	Hardening Parameters		
		E (GPa)	σ_y_ (MPa)	K (MPa)	n
**AB**	Al	59,100 [63]	224.11	77.22	842.32
	Si-rich	66,700	271.38	803	20
**HT1**	Al	59,100 [63]	225.6	69.74	810.02
	Si-rich	66,700	273.73	904.1	13

**Table 3:** Optimized CP parameters for Al determined by inverse analysis. See text for details.

States	${\dot{\gamma }}_{0}(s^{-1})$	$p_{1}$	$p_{2}$	${\hat{\tau }}_{0}^{\alpha }(\mathrm{MPa})$	${\hat{\tau }}^{f}(\mathrm{MPa})$	*h*_0_ (MPa)
AB	0.001	52	2.5	93	190	1117
HT1	0.001	68	2.5	99	128	1152

## Data Availability

The data presented in this study are available upon request from the corresponding author.

## Appendix A

In this section, the importance of accurate texture representation in the RVE on the prediction of the mechanical behavior of PBF-LB/M materials was studied. An ODF was generated based on the crystal symmetry from the experimentally obtained EBSD data using the uniformODF method available in MTEX [33]. Three sets of random orientations were obtained from the created ODF. These orientations were assigned randomly to the RVE grains.

Uniaxial tensile simulations were performed using J2 plasticity and CP models with the help of optimum material parameters (cf. Table 2 and Table 3). Figure A1 shows a big difference between the simulation and the experiment. The random texture also had a similar effect on the prediction of the tensile behavior of the HT1 state of PBF-LB/M AlSi10Mg. Thus, the texture in the RVE must accurately represent the experimentally observed texture.
